# Literacy among tobacco users and healthcare professionals about tobacco harm reduction strategies: a scoping review protocol

**DOI:** 10.3389/fpubh.2025.1518069

**Published:** 2025-01-28

**Authors:** Sajid Iqbal, Sudhanshu Patwardhan, Erika Sivarajan Froelicher, Kainat Asmat, Rubina Barolia

**Affiliations:** ^1^Faculty of Nursing and Midwifery, Shifa Tameer-e-Millat University, Islamabad, Pakistan; ^2^Centre for Health and Edcuation-UK and South Asia, Hampshire, United Kingdom; ^3^Department of Physiological Nursing, University of San Francisco, San Francisco, CA, United States; ^4^School of Nursing and Midwifery, Aga Khan University, Karachi, Pakistan

**Keywords:** tobacco harm reduction, harm reduction, tobacco use, harm reduction strategies, tobacco alternatives, nicotine, electronic cigarettes

## Abstract

**Objective:**

The proposed review identifies and describes the extent of Tobacco Harm Reduction (THR) strategies in Pakistan. It also examines the awareness and understanding of these strategies among healthcare professionals and tobacco users in Pakistan.

**Introduction:**

Tobacco consumption poses a significant threat to human health and is a leading cause of non-communicable diseases. Over the last two decades, the prevalence of tobacco use has increased in low-and middle-income countries (LMICs), which has considerably contributed to the increasing prevalence of Non-Communicable Diseases (NCDs). Tobacco is consumed in various forms, including manufactured cigarettes, hookah/shisha, chewing tobacco, and dipping tobacco. Tobacco use can be reduced and prevented through various strategies adopted by many countries worldwide. One prominent strategy is THR. THR involves the use of safer nicotine products in recommended doses to cope with the symptoms of withdrawal and increase tobacco users’ chances of quitting smoking. In Pakistan, the concept of tobacco harm reduction (THR) is not widely recognized by healthcare professionals and tobacco users.

**Inclusion and exclusion criteria:**

Literature available on Pakistani adults and adolescents (aged 13 years and above) will be included. Literature available as free full text will be included regardless of the publication date. Furthermore, literature available in English or Urdu will be considered eligible. Also, literature available in any form of publication, such as research studies, reviews, organizational websites or blogs, will be considered eligible. Literature that includes Pakistani data mixed with data from any other country will be excluded.

**Methods:**

The literature search will use PubMed, CINAHL, ProQuest Theses & Dissertations, EBSCO Dentistry and Oral Sciences, and PsycInfo databases for online and gray literature. The Preferred Reporting Items for Systematic Reviews and Meta-Analyses (PRISMA) flow diagram will record the included and excluded literature. Two independent reviewers will screen all the retrieved literature. After confirming the eligibility criteria, data from included records will be extracted on an Excel sheet that will record the important characteristics of the literature source and the main findings. Meta-synthesis of collated data will be performed, and the results will be presented as narrative summaries and frequency tables.

**Systematic review registration:**

https://osf.io/dashboard.

## Introduction

Tobacco consumption poses substantial risks not only to the users themselves but also to those around them (1). All types of tobacco use are harmful to health, contributing to a range of non-communicable diseases (NCDs), including various cancers, as well as cardiovascular and respiratory conditions (2). Despite the well-documented adverse effects of tobacco for decades, it continues to be the leading cause of premature mortality and disability worldwide. Each year, tobacco-related health issues result in the deaths of over eight million individuals globally; 80% of these deaths occur in Low and Middle-Income Countries (LMICs) (3, 4). While tobacco use can be prevented or reduced through various strategies, such as Tobacco Harm Reduction (THR), effective awareness and skills regarding the types of THR, their usage, and advising to others are essential for achieving a global impact. Such understanding and skillfulness can significantly impact the appropriate implementation of THR.

The NCDs are currently at the forefront of global research efforts. The World Health Organization (WHO) aims to reduce premature mortality from these diseases by 25% by 2025, while the United Nations (UN) Sustainable Development Goals (SDGs) target a one-third reduction by 2030 (4, 5). Effective tobacco control is crucial for meeting these objectives, as tobacco use contributes to one in six deaths from NCDs (6). Therefore, decreasing the prevalence of tobacco use is vital for managing NCDs and preventing premature fatalities. Considering the devastating health effects of tobacco use, Tobacco Harm Reduction (THR), especially the use of safer nicotine products (SNPs) as an alternative, has demonstrated significant potential in lowering tobacco-related morbidity and mortality (7).

### Tobacco-use prevalence

In 2020, 22.3% of the global population used tobacco, with 36.7% of men and 7.8% of women (2). In the literature, data regarding the actual prevalence of tobacco use worldwide are inconsistent. A survey conducted in 82 LMICs concluded that cigarette smoking was the most common tobacco use in these countries (8). According to the study, 16.5% of the population in these countries were smokers. Smoking is more prevalent in men (33.2%) than in women (3.3%) (8). However, the smoking prevalence in High-Income Countries (HICs) is much lower than that in LMICs. A study conducted in 63 HICs and LMICs found that the prevalence of current smoking in HICs was lesser (14.4%) than that in LMICs (20.95%) (9). This difference is attributed to the numerous initiatives taken effectively in the HICs. For example, HICs such as the United States of America (USA), Canada, and the United Kingdom (UK) introduced electronic cigarettes as a THR strategy that exponentially reduced the prevalence of combustible smoking (10–12). In addition to the effective implementation of antismoking regulations and policies, the widespread introduction of smoking harm reduction strategies is considered vital in reducing smoking prevalence in the HICs. Compared to HICs (e.g., a 13.6% reduction in smoking prevalence between 2006 and 2014 in European Union countries), progress in THR in LMICs is slow (e.g., a 3.7% decrease between 1994 and 2014 in Pakistan) (13).

A study on the Asian population indicates that using tobacco products is considered a cultural norm and a matter of hospitality (14). Tobacco use is a prevalent health risk among the Pakistani population. There is variation in the actual number of smokers in Pakistan; however, it is estimated that 22.2–32% of men and 2.1–6% of women are active smokers in Pakistan (15). More than 22 million people in Pakistan smoke tobacco, accounting for 32% of men and 6% of women. Consequently, tobacco use contributes to 15% of deaths among men and 1% of deaths among women. In such a situation where the tobacco prevalence is not under control, the THR strategy can be a practical approach to reduce the burden of diseases, especially NCDs.

### Tobacco harm reduction strategies

According to the Global State of Tobacco Harm Reduction (16), harm reduction consists of practical policies/regulations and measures to lower health risks by offering safer alternatives to products or substances or promoting less hazardous behaviors. It does not completely eliminate these products or behaviors. Similarly, THR provides the use of safer nicotine or less harmful tobacco products to smokers who want to quit smoking. THR has become a practical option available to them (16). The definition of THR is:

*THR refers to substituting lower-risk nicotine and tobacco products, like nicotine replacement therapy, pharmaceuticals, low-nitrosamine smokeless tobacco products, and e-cigarettes, for the highest-risk tobacco products—cigarettes and other combusted products—for smokers who otherwise cannot or will not quit using nicotine or will not do so soon”* (17).

There are conflicting views on the benefits and risks of tobacco harm reduction (THR) strategies. However, compared with the harm caused by combustible use of tobacco, THR strategies have the potential to reduce harm, mortality, and morbidity without eliminating the use of tobacco and/or nicotine. According to the United States Food and Drug Administration (US FDA), “the available scientific evidence, including long-term epidemiological studies, shows that relative to cigarette smoking, exclusive use of these specific smokeless tobacco products poses a lower risk of mouth cancer, heart disease, lung cancer, stroke, emphysema, and chronic bronchitis” (18).

Alternative tobacco and nicotine products such as nicotine replacement therapy (NRTs), nicotine pouches, electronic nicotine delivery systems (ENDS), electronic cigarettes (e-cigarettes/EC), low-nitrosamine smokeless tobacco, and heated tobacco are self-administered interventions that are less intrusive. These products have the potential to help individuals quit smoking by alleviating withdrawal symptoms (19, 20). These additional strategies support a recommendation toward quitting smoking, usually by offering significantly reduced levels of harmful forms of tobacco such as combusted tobacco, thus lowering health risks due to a substantial decrease in harmful chemical components present (21–23).

On the continuum of risks caused by tobacco, combustible tobacco has been reported to produce the most harmful chemicals, thus posing the highest risk to its users (24). One chemical that poses comparatively less risk than other constituents of tobacco is nicotine. This chemical is the cause of addiction to tobacco products. In case a tobacco user, especially those using combustible tobacco, has a dependency on tobacco and they face difficulty in tolerating the withdrawal symptoms after quitting tobacco, the use of SNPs for a specific period can increase the tobacco quit chances. A systematic review has concluded that the use of NRT increases the chances of tobacco quitting by two to threefold as compared to those who use no THR (25). Despite having nicotine, which is a highly addictive substance, SNPs do not contain numerous harmful chemicals such as tar and carbon monoxide, which cause an array of health problems. It is recommended that these products be used correctly to ensure safety and sustainable tobacco quitting (26). Their sole purpose must be to suppress the urge to use tobacco. On the other hand, using THR, especially in the form of SNPs, is not harmless. These are highly addictive substances and have a negative impact on the cardiovascular, nervous, and reproductive systems of the body, but not to the extent to which tobacco use does. In this regard, only Food and Drug Administration-approved alternatives such as NRT are recommended with adjusted dosage for a specific period of time (26). Hence, THR strategies such as SNPs can substantially reduce harm related to using tobacco. However, they cannot equate to the complete cessation of tobacco and nicotine use, which is the safest approach. In the current review, all alternatives to tobacco use are considered harm reduction strategies, including safer nicotine products such as NRTs (nicotine gums, patches, inhalers), nicotine pouches, ENDS, snus, or any other alternatives with no or minimal tobacco content.

### Problem statement

Recently, two studies were conducted in Pakistan to examine awareness of THR among cigarette smokers and healthcare professionals (27, 28). According to these studies, most smokers and healthcare professionals are unaware of smoking harm reduction strategies. In this milieu, very few (24.7%) smokers in Pakistan intend to quit, and most (97.4%) fail to quit successfully (29).

Misconceptions regarding the health effects of THR measures exist among smokers and healthcare professionals, especially in LMICs such as Pakistan (28, 30). In Pakistan, patients place great trust in the recommendations of their healthcare professionals; therefore, low awareness or misconceptions among these professionals can greatly diminish the likelihood of public adoption of THR. However, a conclusive collation of data regarding THR strategies and their awareness has not been conducted.

### Aims

This scoping review aims to identify the range of THR strategies and the awareness of THR strategies among Pakistan’s healthcare professionals and tobacco users.

### Rationale

Tobacco use and related diseases continue to be major health concerns in Pakistan. In addition, several myths and misconceptions have been found related to tobacco use in the country. For example, a study indicated that many smokers believe that smoking helps alleviate stress, and they depend on it to manage their daily tasks more effectively (31). Also, the researcher frequently heard similar comments from those who use dipping tobacco. Therefore, investing efforts to make tobacco users realize the role of safer nicotine products as a THR strategy is anticipated to be effective in overcoming this deadly behavior.” Thus, there is a clear need to increase smokers’ awareness of THR (32). Second, basic information such as the current range of THR strategies in Pakistan and healthcare professionals’ current level of knowledge and skills in suggesting THR strategies is deemed crucial to developing plans to utilize THR strategies effectively, which will reduce the ever-increasing prevalence of cigarette smoking and associated health adversities. To determine where to begin, it is essential to understand the current situation, which can be achieved by conducting a scoping review of existing literature from Pakistan. The findings of the proposed scoping review will help understand the awareness of THR in Pakistan and the extent to which it is introduced in practice. This will provide a base for further scientific inquiries and interventional studies.

## Methodology

The scoping review will be guided by the Joanna Briggs Institute guidelines, which are underpinned by the framework of Arksey and O’Malley (33, 34). Moreover, the process and documentation of the scoping review will be guided by the Preferred Reporting Items for Systematic Reviews and Meta-Analyses extension for Scoping Reviews (PRISMA-ScR) (35). The review will be carried out in the following nine steps.

### Defining and aligning the objective/s and question/s

This review aims to assess the nature and extent of THR strategies and identify the awareness of THR among tobacco users and healthcare professionals in Pakistan. Hence, the questions were aligned accordingly. What is known from the existing literature about the nature and extent of THR strategies in Pakistan? And, what is the level of awareness among tobacco users and healthcare professionals regarding THR strategies in Pakistan?

### Developing and aligning the inclusion criteria with the objective/s and question/s

Aligned with the objectives and questions of the review, all quantitative and qualitative studies and reviews that address Tobacco Harm Reduction (THR) strategies and/or their awareness among tobacco users, healthcare professionals, or both as their outcomes or primary focus of the report will be included. THR may include NRTs, such as nicotine gums, patches, inhalers, nicotine pouches, ENDS, snus, or any other alternative with no or minimal tobacco content. The included studies will involve populations aged 13 years and above, including adolescents and adults. Only literature in English, English translations, or Urdu will be considered for inclusion. Studies that include data from Pakistan mixed with data from other countries will be excluded.

### Planning approach to evidence searching, selection, data extraction, and presentation of the evidence

The databases PubMed, CINAHL, ProQuest Theses & Dissertations, EBSCO Dentistry & Oral Sciences, and PsycInfo will be extensively searched for literature. The PRISMA flow diagram will record the included and excluded literature.

### Searching for the evidence

**Figure 1:**
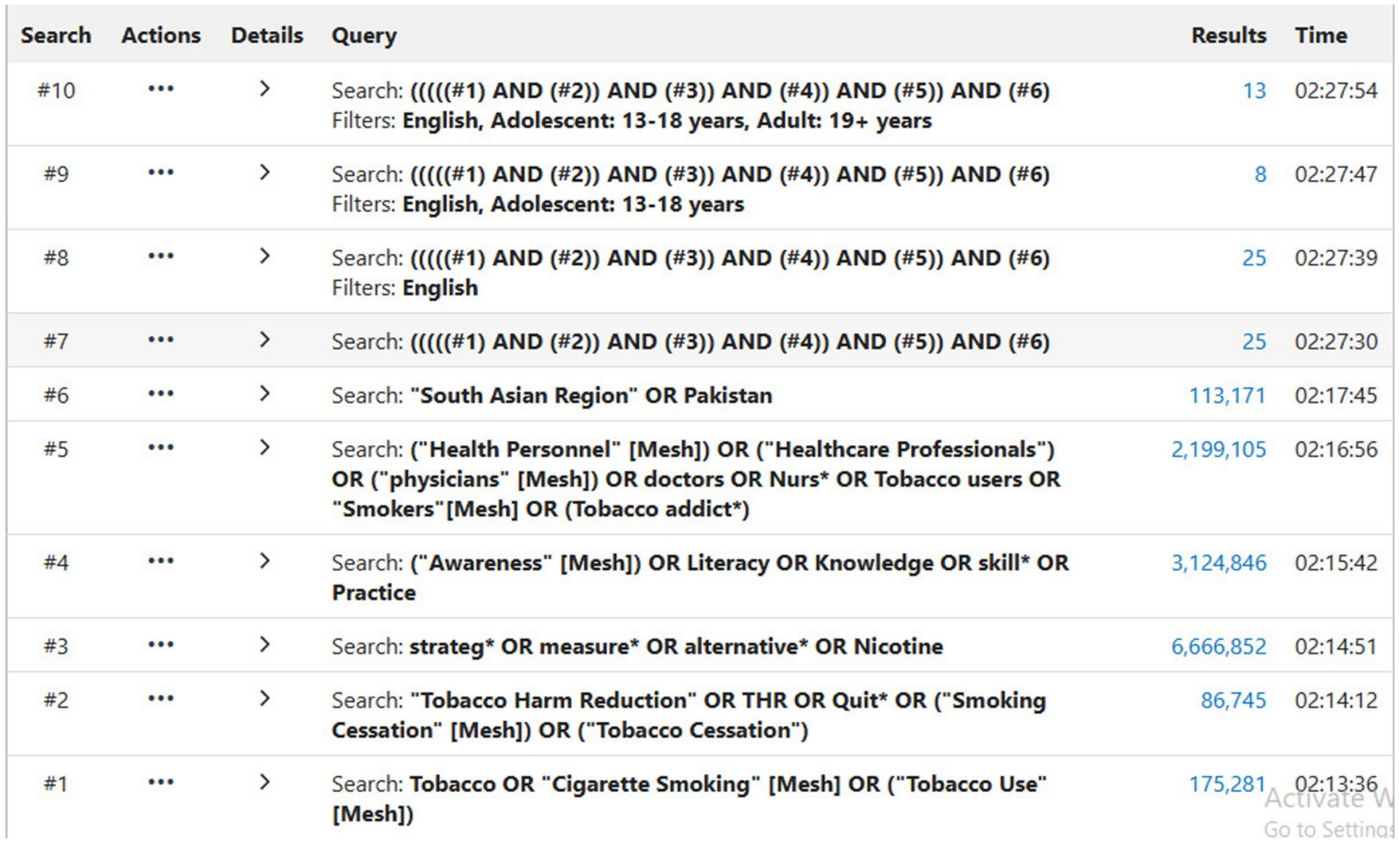
Example of search conducted on PubMed.

After meeting with the librarian, the key terms and their synonyms were selected for the final search. The search was enhanced using MESH terms, Boolean operators, and wildcards. The key terms such as Tobacco harm reduction, E-cigarettes, Nicotine replacement therapy, vaping, smoking cessation, quit, strategies, measures, and South Asian region will be used to develop a nested search syntax as, ((((Tobacco OR “Cigarette Smoking” [Mesh] OR (“Tobacco Use” [Mesh])) AND (“Tobacco Harm Reduction” OR THR OR Quit* OR (“Smoking Cessation” [Mesh]) OR (“Tobacco Cessation”))) AND ((strateg*) OR (measure*) OR (alternative*) OR (Nicotine)) AND (“Awareness” [Mesh]) OR (Literacy) OR (Knowledge) OR (skill*) OR (Practice))) AND (“Health Personnel” [Mesh]) OR (“Healthcare Professionals”) OR (“physicians” [Mesh]) OR doctors OR Nurs* OR Tobacco users OR “Smokers”[Mesh] OR Tobacco addict*)) AND ((“South Asian Region”) OR (Pakistan)) Filters: English, Adolescent: 13–18 years, Adult: 19+ years. An example of a search run on PubMed is shown in Figure 1.

To enrich the data, a manual search of the reference lists of the selected articles will be carried out, and relevant literature will be identified through citation chaining. This will also include manually searching all the literature from organizational websites such as the World Health Organization and Pakistan Tobacco Control Cell, etc. In addition, the primary author will contact other research colleagues or key informants to know about any relevant reports, briefs, or manuscripts in the process of publication. The search for unpublished literature will be assisted by emailing authors of the published article to request any gray literature sources. Additionally, we will utilize the “ProQuest Theses & Dissertations Database,” “OpenGrey,” the Higher Education Commission Research Repository, and WHO country reports on tobacco use to investigate the relevant literature.

### Selecting the evidence

Initially, the two reviewers (SI & KA) will independently review the titles and abstracts of the retrieved records. Based on the inclusion and exclusion criterion, they will decide whether to recommend it for full-text reading. Articles recommended for full-text review will be examined independently by both reviewers. After reviewing the full text, the reviewers will assess the suitability of the articles or any reading materials for inclusion or exclusion. If there is a disagreement in the inclusion or exclusion decision, a third reviewer will resolve the issue.

### Extracting the evidence

Data will be collected using an instrument developed by the researchers. This instrument includes but is not limited to study reference, location, participants’ characteristics (e.g., age, gender, and ethnicity), methodology, research purpose, data collection, and relevant outcomes. This analytical form will be pretested and discussed with the research team to ensure that it captures all relevant information from a selected study.

### Analysis of the evidence

Data from the included records will be analyzed based on the nature of the data. Data from qualitative studies, reviews, and organizational websites will be meta-synthesized through the qualitative content analysis approach of Creswell and Creswell (36). This approach includes five steps: organizing and preparing data, reading and reflecting, data coding, categorizing, and interpretation. Following this approach, the data will be first organized in textual form. In the second step, the data will be thoroughly read and re-read to make sense of the textual data. This will also help comprehend the holistic picture of THR strategies available in Pakistan. In the third step, words, phrases, and sentences relevant to the research questions will be identified, highlighted, and coded to capture their meanings. Based on their nature, THR strategies will be clustered into categories such as oral products, inhaling products, topical products, etc. In the last step, the data will be presented in flow charts supported by textual details.

In addition, data from quantitative literature will be synthesized through descriptive statistics. The data will be pooled in terms of mean scores with standard deviation.

### Presentation of the results

The results of this review will be displayed in the form of chart and narrative summaries. Also, the descriptive data will be presented in tables, relevant diagrams, and in written text.

### Summarizing the evidence

Based on the findings, conclusions will be drawn to clearly and concisely present the overall scenario. Additionally, recommendations will be made to utilize these findings to effectively plan THR strategies in Pakistan.

### Dissemination of the findings

The review findings will be shared with local stakeholders through oral and poster presentations at workshops and conferences. These will also be published in indexed journals. Finally, efforts will be made to obtain opportunities to present work on international platforms, such as participation in the Sigma Theta Tau Conference and the Global Forum on Nicotine.
