# Corrigendum: Literacy among tobacco users and healthcare professionals about tobacco harm reduction strategies: a scoping review protocol

**DOI:** 10.3389/fpubh.2025.1574803

**Published:** 2025-02-21

**Authors:** Sajid Iqbal, Sudhanshu Patwardhan, Erika Sivarajan Froelicher, Kainat Asmat, Rubina Barolia

**Affiliations:** ^1^Faculty of Nursing and Midwifery, Shifa Tameer-e-Millat University, Islamabad, Pakistan; ^2^Centre for Health and Edcuation-UK and South Asia, Hampshire, United Kingdom; ^3^Department of Physiological Nursing, University of San Francisco, San Francisco, CA, United States; ^4^School of Nursing and Midwifery, Aga Khan University, Karachi, Pakistan

**Keywords:** tobacco harm reduction, harm reduction, tobacco use, harm reduction strategies, tobacco alternatives, nicotine, electronic cigarettes

In the published article, there was an error in the **Funding** statement. It originally stated: “The author(s) declare that no financial support was received for the research, authorship, and/or publication of this article.” The correct Funding statement appears below.

